# Colistin Resistance and Management of Drug Resistant Infections

**DOI:** 10.1155/2022/4315030

**Published:** 2022-12-10

**Authors:** Juhi Sharma, Divakar Sharma, Amit Singh, Kumari Sunita

**Affiliations:** ^1^School of Life Science, Jaipur National University, Jaipur, India; ^2^Department of Microbiology, Maulana Azad Medical College, Delhi, India; ^3^Department of Microbiology, Lady Hardinge Medical College, Delhi, India; ^4^Department of Gastroenterology and Human Nutrition, All India Institute of Medical Sciences, Delhi, India; ^5^Department of Microbiology, Central University of Punjab, Bathinda, India; ^6^Department of Botany, Deen Dayal Upadhyay Gorakhpur University, Gorakhpur, Uttar Pradesh, India

## Abstract

Colistin resistance is a globalized sensible issue because it has been considered a drug of the last-line resort to treat drug-resistant bacterial infections. The product of the mobilized colistin resistance (*mcr*) gene and its variants are the significant causes of colistin resistance, which is emerging due to the frequent colistin use in veterinary, and these genes circulate among the bacterial community. Apart from *mcr* genes, some other intrinsic genes and proteins are also involved in colistin resistance. Researchers focus on the most advanced genomics (whole genome sequencing), proteomics, and bioinformatics approaches to explore the question of colistin resistance. To combat colistin resistance, researchers developed various strategies such as the development of newer drugs, the repurposing of existing drugs, combinatorial treatment by colistin with other drugs, a nano-based approach, photodynamic therapy, a CRISPRi-based strategy, and a phage-based strategy. In this timeline review, we have discussed the development of colistin resistance and its management in developing countries.

## 1. Introduction

Antibiotic resistance in Gram-negative pathogens is the greatest threat, given the limited treatment options available for patients with these infections. Ceftazidime-avibactam, meropenem-vaborbactam, and ceftolozane-tazobactam are some combinations of drugs used to combat multidrug-resistant strains of Pseudomonas aeruginosa, Acinetobacter baumannii, and Enterobacteriaceae with metallo-beta-lactamases (MBLs) such as New Delhi MBL (NDM), which are the significant resistant pathogens [1]. In healthcare settings, the emergence of multidrug-resistant (MDR) pathogens such as the so-called “ESKAPEE” group poses a significant challenge that becomes a burning concern due to the high levels of antibiotic resistance. Enterobacteriaceae caused an overreliance on last-resort antibiotics, e.g., colistin/polymyxins due to the emergence and rapid spread of particular strains of carbapenemase-producing bacteria [2].

Colistin is an antimicrobial agent extracted from *Paenibacillus polymyxa,* which comes under the class polymyxin group. Class polymyxin antibiotic contains five polymyxins: A, B, C, D, and E, of which polymyxin E (colistin) and polymyxin B are clinically relevant [3]. In humans, colistin sulfate (CS) is used for oral and topical administration, while colistin methane sulfonate (CMS) sodium is used for parenteral treatment. It is one of the last resorts for antibiotics that are used to treat drug-resistant bacterial infections [4]. In addition, colistin is a popular drug for the veterinary, not only to treat infections caused by Enterobacteriaceae but also as a growth promoter and a protective agent [5]. In recent years, colistin has been considered the drug of last resort in the case of infections by multidrug-resistant Gram-negative bacteria and has begun to be used in humans, notably by carbapenemase-producing Enterobacterales, *Pseudomonas aeruginosa,* and *Acinetobacter baumannii* [6–9].

### 1.1. Mode of Action

The outer cell membrane is the major site of colistin action in Gram-negative bacteria. In the outer membrane, colistin binds to lipopolysaccharides through electrostatic interaction between the *α*, *γ*-di-aminobutyric acid of colistin and the phosphate groups of the lipid A region of lipopolysaccharide (LPS). From the phosphate groups of membrane lipids, divalent cations (Ca^2+^ and Mg^2+^) were displaced by colistin [10, 11]. The phospholipid bilayer in Gram-negative bacteria loses its stability due to the action of colistin, which adds hydrophilic groups to the fatty acid chains, changing its integrity, failing to maintain cellular content, and leading to cell lysis [9]. The disruption of LPS may cause increased permeability of the outer membrane and leakage of intracellular contents, ultimately leading to cell death [7, 12, 13]. Colistin also exerts antiendotoxin activity to prevent endotoxin-mediated shock [12].

### 1.2. Use of Colistin in Patients Care

In recent years, there is a growing interest in deprecating the antibiotic, colistin, with the scarcity of antimicrobials as the available options. A guide for colistin therapy and its optimal clinical use is provided by international consensus recommendations [14]. Colistin is often the last line of defense against multidrug-resistant Gram-negative bacteria such as carbapenemase producers in the Enterobacterales [4, 15], *Pseudomonas spp.*, and *Acinetobacter spp*. It is used particularly in critical clinical conditions such as bacteremia/sepsis and pneumonia associated with mechanical ventilation (VAP) in the intensive care unit. For other clinical conditions, colistin is seen as an alternative treatment such as urinary tract infections, osteomyelitis, joint infections, meningitis, pneumonia, infections of the gastrointestinal tract, pyoderma, soft tissue infections, eye infections, and ear infections. Because of its nephrotoxicity, colistin should be administered carefully, with dose correction as needed, and tight surveillance in patients with renal impairment. A synergetic activity of the combinatorial treatment of colistin with ceftazidime, rifampicin, and amikacin has been reported in *Pseudomonas*, as well as infections caused by MDR *Pseudomonas aeruginosa* [12].

### 1.3. Frequent Use of Colistin in Veterinary: A Plausible Cause of Drug Resistance

At present, colistin is still widely used as an antibiotic in veterinary medicine, mostly in pigs, for the oral treatment of intestinal infections caused by Enterobacterales [16]. Colistin is most frequently used in food-producing animals such as pigs and poultry to control intestinal infections [17]. Overall, higher proportions of resistant isolates are found in treated pigs with colistin as compared with untreated [16]. Additionally, to promote the growth of fish, colistin sulfate has also been used in the seafood industry. Extensive uses of colistin in animals create high selective pressure in the veterinary environment [18]. In calves, colistin is also used orally for the treatment of gastrointestinal diseases, which are majorly caused by Gram-negative bacteria. Although in oral treatment, colistin is generally used as monotherapy [5], in the market, with the sulfate salt form, there are some pharmaceutical forms that also allow combined therapy. Association with other antimicrobials like beta-lactams is the most common and involves mainly amoxicillin [19]. As a general attitude with all antimicrobials, especially with colistin, the veterinary surgeon should ensure that the prescribed antimicrobial is applied strictly for the treatment of sick animals according to recommended protocols. Frequent use of colistin could be a significant factor to trigger the resistance in bacteria and responsible for the emergence and circulation of the *mcr*1-10 genes among the bacterial communities.

### 1.4. Colistin Resistance

Several studies showed that the prevalence of colistin resistance in Enterobacteriaceae has increased rapidly. Clinicians should be alert due to the development of colistin resistance through mutation or adaptation mechanisms among MDR bacteria. The scientific community, experts, government authorities, and public-private consortia have urged for a reduction in colistin usage, commending its prescription only for the treatment of infections as it has been considered a last resort drug.

Colistin usually disrupts the structure of cell membrane phospholipids and increases cell permeability by a detergent-like action, causing cell death. Colistin resistance is predominantly achieved through a reduction of the electrostatic attraction between colistin and the Gram-negative outer membrane which is due to the addition of cationic phosphoethanolamine or 4-amino-4-deoxy-L-arabinose (L-Ara4N) moieties to phosphate groups on the lipid-A component of LPS and reduces the net anionic charge of the cell surface. The mutation leads to the addition of cationic groups to lipid A which weakens the binding of polymyxins [20, 21]. Transposable genetic elements (mostly plasmids with the *mcr* genes) are the major cause of bacterial colistin resistance in the microbial world. To date, ten variants of the mobilized colistin resistance genes (*mcr*), *mcr*1-10, have been identified. Apart from *mcr*, (major responsible factor for plasmid-borne colistin resistance) few other chromosomal genes, *mgrB PhoP-PhoQ*, *PmrA-PmrB* (two-component regulatory systems) mutations, biofilm, and efflux pump have been involved in the colistin resistance due to the deregulation or loss of function. Colistin resistance mechanisms remain unknown for some bacterial species, but several molecular mechanisms have been put forward to explain the mechanisms of colistin resistance, but still, our knowledge regarding resistance is fragmentary.

### 1.5. Intrinsic Resistance Mechanisms by Chromosomal Gene

Naturally occurring resistance to polymyxins is linked to the constitutive expression of the *arnBCADTEF* operon and/or the *eptB* gene (chromosomal gene), causing the addition of phosphoethanolamine (pEtN) and/or 4-amino-4-deoxy-L-arabinose (L-Ara4N) cationic groups to the LPS in *P. mirabilis* and *S. marcescens*. This modification increases the charge on LPS (the initial target of the polymyxins), which decreases polymyxin binding and leads to resistance [21]. A recent study revealed that colistin exposure enhances the expression of *eptB* in colistin-resistant *E. coli* coharboring *mcr*-1 [22].

### 1.6. Acquired Resistance Mechanisms

#### 1.6.1. Chromosomal Gene-Mediated Resistance

Acquired resistance to polymyxins has been identified in several genera of the Enterobacteriaceae, such as *Klebsiella, Escherichia, Enterobacter*, and *Salmonella*. A single transferable mechanism of resistance has been identified so far, with most of the resistance mechanisms being encoded chromosomally. Similar to what is observed in strains that are naturally resistant to colistin, the addition of cationic groups (L-Ara4N and pEtN) to the LPS is responsible for the acquisition of colistin resistance in Enterobacteriaceae. Colistin resistance thus is the result of modification of LPS via chromosomal genes and operons encoding enzymes that have a direct role in LPS modification, such as the *pmrC* and *pmrE* genes and the *pmrHFIJKLM* operon; regulatory two-component systems (TCSs) *PhoP-PhoQ*, *PmrA-PmrB*, as well as *crrA-crrB,* which regulate the *PmrA-PmrB* system. The *mgrB* gene, a negative regulator of *PhoP-PhoQ*, while *crrA-crrB regulate PmrA-PmrB,* while plasmid-mediated *mcr* genes, c*px,* and *rcs* lead to the upregulation of capsule biosynthesis, the activator of the efflux pump, and regulating the *PhoP-PhoQ* system, respectively [23].

Various PETN-coding genes, such as *eptA* (pmrC), *eptB* (pagC), and *eptC* (cptA), are able to add PETN to dissimilar sites of LPS. Alteration of the phoP-phoQ genes has been recognized in *K. pneumoniae* and *E. coli*, leading to attained colistin resistance [24]. Mutations or disruptions in the *mgrB* gene have been reported as a potential reason for colistin resistance; however, *mgrB* inactivation is the greatest mechanism for colistin resistance in *K. pneumoniae* and *K. oxytoca*. Amino-acid substitutions of the CrrB protein result in increased autophosphorylation of this protein, which consequently leads to colistin resistance [25].

### 1.7. Plasmid-Mediated Resistance

Transposable genetic elements or *mcr* genes are the major cause of bacterial colistin resistance, and to date, ten variants of the *mcr*1-10, have been identified. In 2015, the first plasmid-mediated colistin resistance was detected in the *E. coli* strain of Chinese animals [26]. Generally, low-level colistin resistance with a minimum inhibitory concentration (MIC) in the range of 2–8 mg/l is characterized in *E. coli* strains with the *mcr*-1 gene. The higher mutation rate in the chromosomal polymyxin resistance cascade genes produced higher MIC values (≥64 mg/l) caused by the expression of the *mcr*-1 gene in *E. coli* [27]. Another novel plasmid-mediated colistin resistance gene, known as *mcr* 2, is in E. coli [28]. After this *mcr*3 and *mcr*4 genes were discovered [29, 30]. Finally, in July, from *Salmonella paratyphi* B a new gene of the *MCR* family was carried in transposons instead of plasmids [17]. In addition, in 2018 three mobile colistin-resistance genes (*mcr*6, *mcr*7, and *mcr*8) were discovered. In a patient (Washington State), the *mcr*-9 gene, a novel *mcr* homolog detected in the MDR colistin-susceptible *Salmonella enterica serovar Typhimurium* strain, was isolated [31]. According to the European Committee on Antimicrobial Susceptibility Testing (EUCAST), this strain was phenotypically sensitive to colistin with a MIC value of 2 mg/l. In vitro expression of the cloned *mcr-9* gene in the *E. coli* NEB5*α* strain confirmed colistin resistance. Recently, Wang et al. [32] isolated *mcr*10 from a patient in China.

### 1.8. Biofilm-Mediated Colistin Resistance

Biofilm-mediated antibiotic resistance is also a well-known phenomenon. Correlation between the biofilm-forming ability regulated by genes/proteins and colistin resistance in bacteria has been shown in several studies, which depicted their inter-relationships with the resistance phenotype [33–35]. Several bacterial species produced biofilms, which promote tolerance to antimicrobials and hinder their penetration. Recently, a study was done to establish a possible relationship between biofilm-forming capacity and the antibiotic-resistant phenotype in clinical *Acinetobacter baumannii* [34]. Biofilm formation is positively correlated with the differential expression of many relevant virulence factors, including flagellar, fimbriae, pili, surface proteins, and the production of poly-*β*-(1-6)-N-acetylglucosamine (PNAG) and acyl-homoserine lactone (AHL) signal molecules [35–37]. Bacteria embedded in deeper layers of the biofilm seldom come into contact with antibiotics, due to the inability of these drugs to adequately penetrate into its deeper layers; this results in a 10–1000-fold higher MIC as compared to planktonic cells [35–37]. Most recently, our proteomics-based study on colistin-resistant *E coli* revealed that a panel of differentially expressed proteins, which could be unveiled the mechanism of colistin resistance [33]. This study also suggested that these proteins and their pathways could be used to develop novel therapeutics against colistin-resistant infections. An alteration in the *mgrB* gene (a negative regulator) in the resistant strains could be potentially due to increased expression of both biofilm-forming and quorum-sensing genes. Mutations in *mgrB* could lead to the dysfunctionality of the *phoP-phoQ *two-component system which is further accountable to colistin-induced resistance by cumulative expression of biofilm-forming and quorum-sensing genes [38].

### 1.9. Efflux Pumps-Mediated Resistance

The role of efflux in colistin resistance is not well understood, but studies have suggested the involvement of efflux pumps in colistin resistance [39–41]. Lin et al. [40] suggested that EmrAB efflux pumps contributed to colistin resistance in *Acinetobacter baumannii.* The addition of low doses of the efflux pump inhibitor carbonyl cyanide m-chlorophenylhydrazone (CCCP) into the medium decreased the MICs for resistant strains (128 to 512-fold reductions) and partially or completely inhibited the growth of resistant subpopulations [41]. However, this observation should be considered with caution owing to the nonspecific effect of CCCP on efflux systems and its likely wider impact on bacterial metabolism. Combinatorial use of a *MarR* inhibitor (enhancer of colistin binding) and an efflux pump inhibitor (reducer of colistin extrusion) was suggested to restore colistin sensitivity in colistin-resistant strains of *E. coli* in vitro and in vivo [39].

### 1.10. Strategies to Combat Colistin Resistance

Colistin has been considered a last-line drug to treat drug-resistant infections. Nowadays, worldwide, the rates of colistin resistance vary between bacterial species such as 3 and 28% for *A. baumannii* and 2.8 and 10.5% for *K. pneumoniae.* The emergence of colistin-resistant microbes/bed bugs and their management are significant concerns globally. Worldwide, it is reported that he rise in consumption of colistin leads to increased cases of colistin-resistant multidrug-resistant strains and threatens to return clinicians and patients to a “preantibiotic era.” Therefore, it creates a therapeutic challenge to manage the colistin-resistant strains that produce lactamases. In this context, the development of new molecules/drugs, repurposing of the existing drugs, combination treatment by colistin with other drugs, and new promising strategies (nano-based strategy, photodynamic therapy, and CRISPRi based strategy, Phage based strategy) are potential to combat the colistin-resistant deadly bed bugs.

Researchers are continuously trying to develop new molecules/drugs with a novel mode of action and potentially effective against the varieties of MDR organisms. Fluopsin C, a bioactive secondary metabolite (a metal-containing antibiotic) extracted from *Streptomyces* and *Pseudomonas species*, showed effective antimicrobial activities against Gram-positive, Gram-negative, and drug-resistant bacteria [42, 43], Sharma 2020). Terrein is another purified metabolite extracted from the fungus that showed better antimicrobial activities against *S. aureus, A. hydrophila*, *E. Faecalis,* and other microbes [44]. Therefore, we suggested that after clinical approval these molecules could be used as potential drugs against colistin-resistant bacteria.

Classical combinations between colistin and other antimicrobial agents have been reported to treat drug-resistant bacterial infections which are popularly called combinatorial therapy [45]. Other antibiotics including tigecycline, meropenem, gentamicin, or fosfomycin are often used in combination with colistin [46]. A study has reported that a patient with ventilator-associated pneumonia (VAP) caused by colistin-resistant bacteria was successfully managed using a combination therapy of colistin, vancomycin, and rifampicin [47]. The effects of this combination therapy are to be confirmed by a randomized clinical trial that has been underway [48].

**Figure 1:**
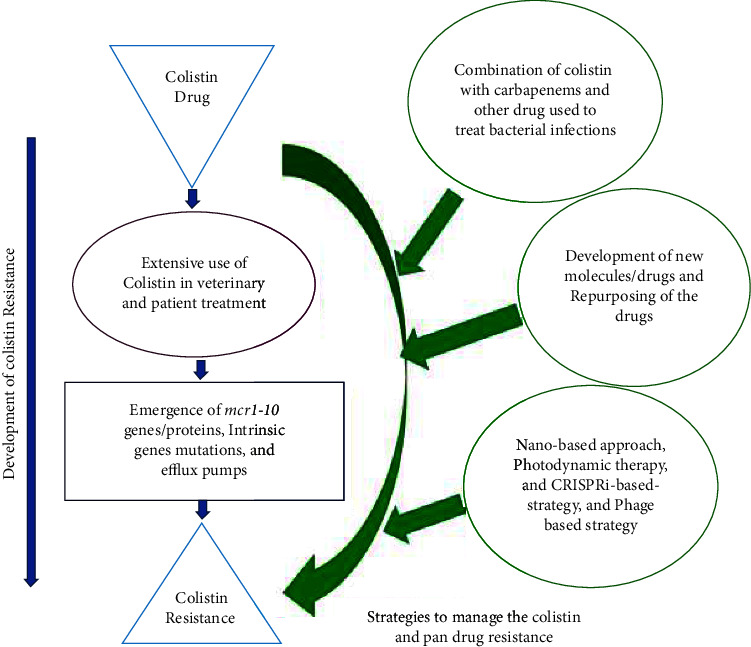
Development of colistin resistance and combating approaches.

Investigating the new uses of already existing drugs defined as “repurposing drugs,” has gained attention, as has using them to manage colistin-resistant strains [49]. Ellipticine, a natural alkaloid, and its analogs were initially reported as an anticancer agent [50]. However later its antibacterial effectiveness was also accessed against colistin-resistant *E. coli* and considered a potent molecule to combat the deadly bad bugs [51]. Research suggested niclosamide (an anthelmintic drug) could be repurposed in the combination with colistin to treat colistin-resistant Gram-negative bacillary infections [52]. Repurposing of anthelmintic nonantibiotic molecules with a colistin combination has been shown to combat colistin-resistant Gram-negative bacteria [53]. PFK-158, an antitumor drug, has been repurposed and showed a synergistic effect with colistin against colistin-resistant Enterobacteriaceae [54].  Figure 1 indicates the probable development of colistin resistance in developing countries and combating approaches to managing these deadly infections.

In an in vitro study, researchers used the CRISPR/Cas9 approach to remove plasmid (having *mcr*-1 gene) in a stepwise manner or simultaneously remove multiple plasmids in one step. Therefore, this approach could be used to delete multiple gene copies by using only one sgRNA. However, caution should be taken to avoid unwanted recombination events [55]. Most recently, Khambhati et al. [56] suggested that CRISPR-assisted phage genome engineering be employed to generate phage variants, which could combat drug resistance [56].

The nano-based strategy has been reported to have the potential to combat drug-resistant infections. Researchers reported that the MIC of colistin and AgNPs against the pan-drug-resistant *Acinetobacter baumannii* was higher as compared to the combination of colistin and AgNPs (Col-AgNPs). Therefore, this combination showed a synergistic effect and led to a reduction in MIC [57]. Col-AgNPs exhibited higher cell survival than AgNPs and colistin which could enhance the antimicrobial activity and cell biocompatibility.

Photodynamic therapy (PDT) based research suggests that it could be used as an alternative strategy to treat drug-resistant infections. In an in vitro study, Pourhajibagher et al. [58] evaluated the effect of PDT along with colistin on pan-drug-resistant *Acinetobacter baumannii.* They observed that PDT along with colistin showed a synergistic effect and eliminated all the pan-drug-resistant *Acinetobacter baumannii* by decreasing the colistin MIC by more than 11-fold as compared to PDT alone [58]. Another study has shown the effect of PDT therapy against the ompA virulence genes expression in colistin-resistant *Acinetobacter baumannii* and found that the overexpression of ompA could assist in more penetration of the drug [59].

Phase therapy has also re-emerged as a novel strategy for combating bacterial infections and antibiotic resistance. By measuring zeta potentials, Hao et al. [60] observed that at pH7 (neutral) phage particles were negatively charged, and colistin-resistant bacteria had less negative zeta potentials as compared to the wild type. Therefore, the decreased negative surface charge of colistin-resistant cells leads to a decrease in the electrostatic repulsion between the bacteria and phage, which promotes phage adherence followed by subsequent infection [60]. A study suggests that combinations of phage Phab24 with colistin lead to changes in envelope architecture that decreased the resistance in colistin-resistant *Acinetobacter baumannii* [61]. This decrease in antibiotic resistance is a direct consequence of the phage-resistance mechanism, and could potentially be exploited in the clinical setting.

## 2. Discussion

**Figure 2:**
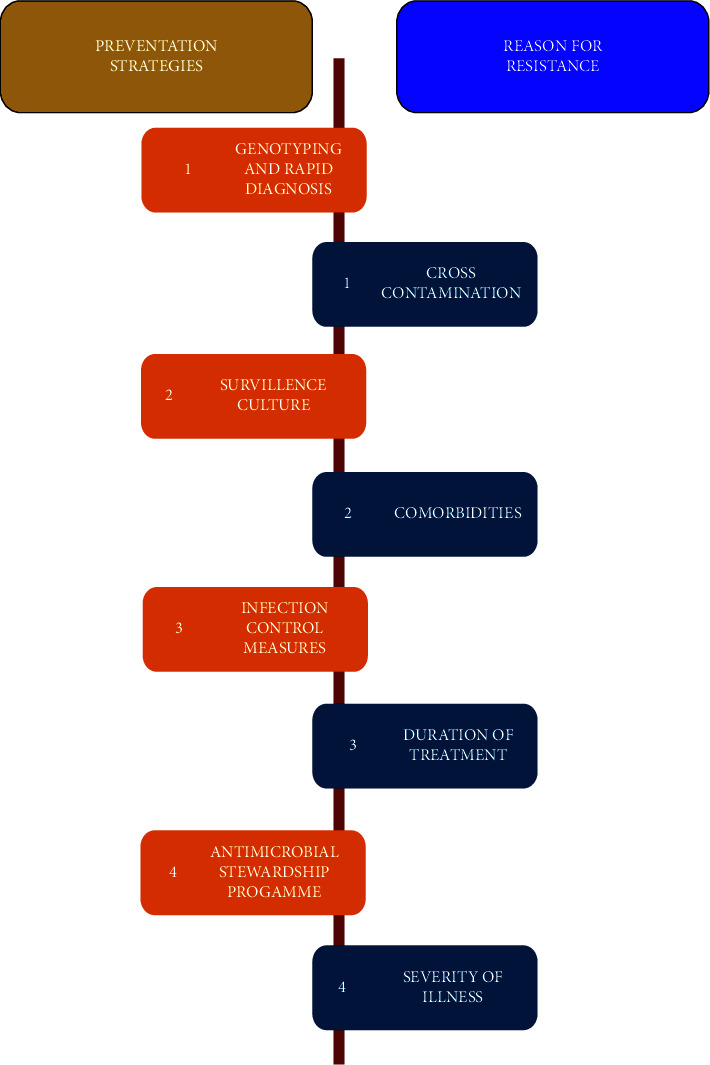
Prevention strategies and plausible factor for colistin resistance.

The development of carbapenem-resistant Enterobacteriaceae (CRE) has become a significant challenge [62–65], which leads to the use of colistin across the globe including in South Asian Countries [13, 66]. The worldwide spread of carbapenemase-expressing Enterobacteriaceae needs immediate efforts toward early detection and infection control measures as it signifies a substantial threat to public health [66]. In the current therapeutic scenario, colistin is used as the last alternative antimicrobial against MDR and PDR (pan-drug-resistant) Gram-negative infections [11, 13, 67]. The genetic basis of colistin resistance in *Acinetobacter baumannii* is being explored [68–70], but it still remains unclear whether similar mechanisms are associated with colistin resistance in carbapenem-resistant Enterobacteriaceae. Different bacterial species have developed resistance due to the inappropriate use of colistin. Inappropriate dosing of colistin might tip to colistin resistance among carbapenem-resistant *K. pneumoniae* strains and other microbes, while optimal dosing programs have not been determined for colistin [71]. Prevention strategies play an important role in the management of the emergence of colistin-resistant bad bugs (Figure 2).

Colistin resistance is also seen in clinical, food, and food animal isolates; this resistance is increasing gradually and is considered a noteworthy problem worldwide. Colistin resistance is due to the use of colistin in animal farms as an animal food preserver and growth promoter. The people working on animal farms and related industries have a serious threat as they may get infections easily from animals. Such isolates may also spread via water sources as well as act as a reservoir in the environment. Resistance to this last-line drug causes enormous difficulties in the treatment of patients infected with MDR and XDR isolates. Colistin resistance dominance among clinical Gram-negative isolates was beneath 6%. Dalmolin et al. [72] findings are analogous to the Gram-negative finding as the average dominance of colistin resistance was around 7% among *E. coli, Klebsiella pneumoniae*, and *Enterobacter species* [72]. Many colistin-resistant clinical isolates were also resistant to a wide variety of antimicrobial agents, comprising penicillins, cephalosporins, monobactams, carbapenems, aminoglycosides, quinolones, nitrofurans, and etc. Colistin-resistant *K. pneumoniae* from Pakistan was found to be resistant to twenty-three antimicrobial agents from ten antimicrobial groups except for tigecycline [68]. Likewise, colistin-resistant *Acinetobacter* isolates were not accountable to imipenem, meropenem, ampicillin-sulbactam, ciprofloxacin, gentamicin, and amikacin resistance [73]. The animal isolates have a higher incidence of colistin resistance with reference to clinical isolates as up to 69% of *E. coli* isolates recovered from milk were resistant to colistin. Amongst various colistin-resistant bacterial strains obtained from various animal samples, *E. coli* was the major isolate.

Mobilized colistin resistance (*mcr*) gene or *mcr*-harboring bacterial isolates have been reported from six continents (Asia, Europe, Africa, North America, South America, and Oceania) and over 27 bacterial species since the first isolation of *mcr*-1 in China [26], the numbers of reports have been increasing due to long term use of colistin/polymyxins in veterinary as a medicine. It is worth noting that before 2005, the *mcr*-harboring isolates were not identified in any reports, but most of the isolates reported in the last decade were *mcr*-positive historic isolates. The major suggested causes are the importation of food from infested countries like Japan and Tunisia [74, 75], the over-prescription of colistin in human medicine to treat exceedingly resilient clinical pathogens in Argentina [76], global trade, and travel to countries with high or unknown prevalence like Canada [77], USA [78], and Japan [79]. To date, quite a few other *mcr* gene variants have been recognized, including *mcr*-2, -3, -4, -5, -6, -7, and -8, which share 81%, 32%, 34%, 36%, 83%, 35%, and 31% amino acid sequence personality, separately, with *mcr*-1 [80]. The *mcr*-9 is closely related to *mcr*-3, as reported [31]. In a recent study, it was described that the recently found *mcr*-9 gene in Salmonella typhimurium, from a clinical isolate in the USA, was capable of conversing phenotypic resistance to colistin in Enterobacteriaceae, making it a significant concern to the public health [31]. Andrade et al. [81] summarized the colistin action and its drug resistance mechanisms [81–83]. Recently a study explored the bunch of proteins has been involved in colistin resistance by proteomic and bioinformatics [33], which could be employed to explore future research questions in the field of colistin resistance.

## 3. Conclusion and Future Prospects

Nowadays, colistin resistance has become the greatest issue to treat globally. Various studies have proven this resistance in several bacterial species worldwide. In view of the fact of its ability to pass on horizontally from one bacterium to another, between animals and humans; therefore, the *mcr*1-10 genes were acknowledged as the major responsible factor for colistin resistance. Apart from *mcr* genes, some chromosomal genes, *mgrB PhoP-PhoQ, PmrA-PmrB*, biofilm formation, and efflux pump have been considered as potential factors for colistin resistance. Most of the resistant bacteria were also featured as being MDR with or without colistin resistance. To combat this deadly situation various strategies have been employed. Researchers have developed newer molecules/antibiotics, with better effects and more tolerance than colistin, using antibiotic groupings with different antibiotics, or even with nonantibiotic molecules which have become a novel substitute for colistin. We cannot completely rely on the discovery of newer antibiotics because after the implication of antibiotics bacteria developed resistance rapidly. Repurposing of the drugs, combinatorial treatment by colistin with other drugs, other promising strategies such as nano-based strategy, photodynamic therapy, CRISPRi based strategy, and Phage-based strategy, etc., potential to combat the colistin resistance. The combination of meropenem and colistin has shown a synergistic effect against antibiotic-resistant Gram-negative bacteria and has the potential to reduce the development of resistance. From this focused review, we suggested that the combination of drugs (like meropenem and colistin) and other strategies (nano-based strategy, photodynamic therapy, CRISPRi-based strategy, and Phage based strategy) could be employed to combat drug-resistant bad bugs and a possible option to manage this greatest issue [83–86].

## Data Availability

All data are included in the submitted text and tables.
